# The role of computed tomography features in assessing response to neoadjuvant chemotherapy in locally advanced gastric cancer

**DOI:** 10.1186/s12885-023-11619-2

**Published:** 2023-11-27

**Authors:** Chengzhi Wei, Yun He, Ma Luo, Guoming Chen, Runcong Nie, Xiaojiang Chen, Zhiwei Zhou, Yongming Chen

**Affiliations:** 1 Department of Gastric Surgery, State Key Laboratory of Oncology in South China, Guangdong Provincial Clinical Research Center for Cancer, Sun Yat-sen University Cancer Center, Guangzhou, 510060 P. R. China; 2 Department of Medical Imaging, State Key Laboratory of Oncology in South China, Guangdong Provincial Clinical Research Center for Cancer, Sun Yat-sen University Cancer Center, Guangzhou, 510060 P. R. China

**Keywords:** Gastric cancer, Computed tomography, Neoadjuvant chemotherapy, Pathological response, Tumor regression grade

## Abstract

**Objective:**

To compare the computed tomography (CT) images of patients with locally advanced gastric cancer (GC) before and after neoadjuvant chemotherapy (NAC) in order to identify CT features that could predict pathological response to NAC.

**Methods:**

We included patients with locally advanced GC who underwent gastrectomy after NAC from September 2016 to September 2021. We retrieved and collected the patients’ clinicopathological characteristics and CT images before and after NAC. We analyzed CT features that could differentiate responders from non-responders and established a logistic regression equation based on these features.

**Results:**

We included 97 patients (69 [71.1%] men; median [range] age, 60 [26–75] years) in this study, including 66 (68.0%) responders and 31 (32.0%) non-responders. No clinicopathological variable prior to treatment was significantly associated with pathological response. Out of 16 features, three features (ratio of tumor thickness reduction, ratio of reduction of primary tumor attenuation in arterial phase, and ratio of reduction of largest lymph node attenuation in venous phase) on logistic regression analysis were used to establish a regression equation that demonstrated good discrimination performance in predicting pathological response (area under receiver operating characteristic curve 0.955; 95% CI, 0.911–0.998).

**Conclusion:**

Logistic regression equation based on three CT features can help predict the pathological response of patients with locally advanced GC to NAC.

## Introduction

Neoadjuvant chemotherapy (NAC) is recommended in patients with potentially resectable gastric cancer (GC) with clinical T3, T4, or node-positive, in order to downstage the tumor and treat micrometastatic tumors. This modality opens more possibilities for maximum systemic therapy delivery [1, 2]. Nevertheless, not all patients with GC are equally responsive to NAC. Differentiating between potential responders and non-responders is helpful in evaluating the sensitivity of patients to chemotherapy, but also in adjusting the comprehensive treatment strategy timely.

Currently, the pathological tumor regression grade (TRG) is a fundamental tool to evaluate the response to NAC and predict patient prognosis. The prognostic role of multiple TRG systems in GC has been confirmed by published data [3–5]. Considering that the TRG is confirmed invasively and mostly after surgery, the use of preoperative radiographic features to accurately predict TRG is important. Among all imaging evaluation systems for the efficacy of NAC, the Response Evaluation Criteria in Solid Tumors (RECIST) based on the measurement of the largest tumor diameter is the most widely used in clinic. Unfortunately, a recent study has shown that RECIST 1.1 was in poor agreement with TRG and was less capable of predicting survival of patients with GC than TRG [6]. How to accurately and conveniently screen pathological responders before surgery remains an unsolved problem.

Recently, it was found that reduction in GC lesion thickness could help predict pathological complete response [7]. Moreover, post-NAC decreased tumor attenuation has been reported to be associated with improved clinical outcome of GC [8]. Therefore, we hypothesized that computed tomography (CT) features including the reduction in the tumor thickness and attenuation of the primary lesion may be potential predictors of TRG. Here, we attempted to integrate these easily accessible CT features to establish a multivariate logistic regression equation aimed at individualized prediction of response to NAC for locally advanced GC, in order to accurately differentiate responders from non-responders.

## Patients and methods

### Ethical approval

This study complied with the ethical standards of the Helsinki Declaration and was approved by the ethics committee of the Sun Yat-sen University Cancer Center. The ethical approval number is B2022-673-01. The present study evaluated 97 patients with locally advanced GC who underwent gastrectomy after NAC from September 2016 to September 2021 at the Sun Yat-sen University Cancer Center. All methods were performed in accordance with the relevant guidelines and regulations.

### Patients

We retrieved and collected clinicopathologic data and enhanced CT images before and after NAC of 97 patients subjected to radical gastrectomy after NAC. Were included, patients with (1) gastric adenocarcinoma confirmed by endoscopic biopsy; (2) non-metastatic locally advanced GC (cT_3 − 4b_N_1−3_M_0_) determined by CT examinations; (3) radical gastrectomy after standardized NAC; and (4) tumor response confirmed on postoperative pathology. The main exclusion criteria were (1) other types of gastric malignancies (including neuroendocrine tumors); (2) non-resected or inoperable cases; (3) concomitant immunotherapy or radiation therapy; and (4) low-quality CT imaging that is difficult to perform measurements.

### Neoadjuvant chemotherapy and pathological response assessment

All patients uniformly underwent 2 to 4 cycles of fluorouracil in combination with platinum-based NAC. The dose of NAC for each patient was determined by the treating oncologists, as per the National Comprehensive Cancer Network (NCCN) guidelines. The patients underwent gastrectomy about 3 weeks after NAC.

The resected specimens were histopathologically evaluated by pathologists with more than 10 years of experience in tumor assessment. We used the TRG scoring system recommended by NCCN guidelines [9] to evaluate NAC response. Complete response (TRG 0) was defined as tumor samples without viable cancer cells. Poor or no response (TRG 3) was defined as extensive residual cancer with no evident tumor regression. Near complete response (TRG 1) was defined as tissues with single cells or rare small groups of cancer cells and partial response (TRG 2) was defined as residual cancer cells with evident tumor regression but more than single cells [9]. Moreover, patients with TRG 0–2 were considered responders to NAC, whereas those with TRG 3 were considered non-responders.

### CT protocol

All patients underwent abdominal CT before and after NAC using the 64-slice spiral CT system (LightSpeed VCT, GE Medical Systems, Milwaukee, WI, USA). After a fast of at least 6 h, the patients were asked to drink 500–1000 ml of water to fill the stomach to a lowtension state before the examination. Enhanced CT images were obtained after acquisition of non-enhanced CT images by intravenous administration of 2 mL/kg of body weight of contrast media (Ultravist 370, Bayer Schering Pharma, Berlin, Germany), at a rate of 3.0 or 3.5 ml/s. The arterial phase and venous phase scans were initiated at 35 and 65 s after the start of contrast injection, respectively. The main acquisition parameters were 120 kVp, 150–190 mAs of automatic adjustment, and a collimation of 1.25 mm. CT images were reconstructed with 2.5 mm thickness in multiple layers.

### Image analysis

Image analysis was performed jointly by two experienced radiologists (He and Luo) using the Picture Archiving and Communication System (Carestream, Canada). They were only aware of the patients’ diagnosis of gastric adenocarcinoma and were completely blinded to the pathological TRG and stage. Any discrepancies in measurements were judged by rigorous discussion. All measurements were performed three times before and after NAC and averaged respectively.

**Fig. 1:**
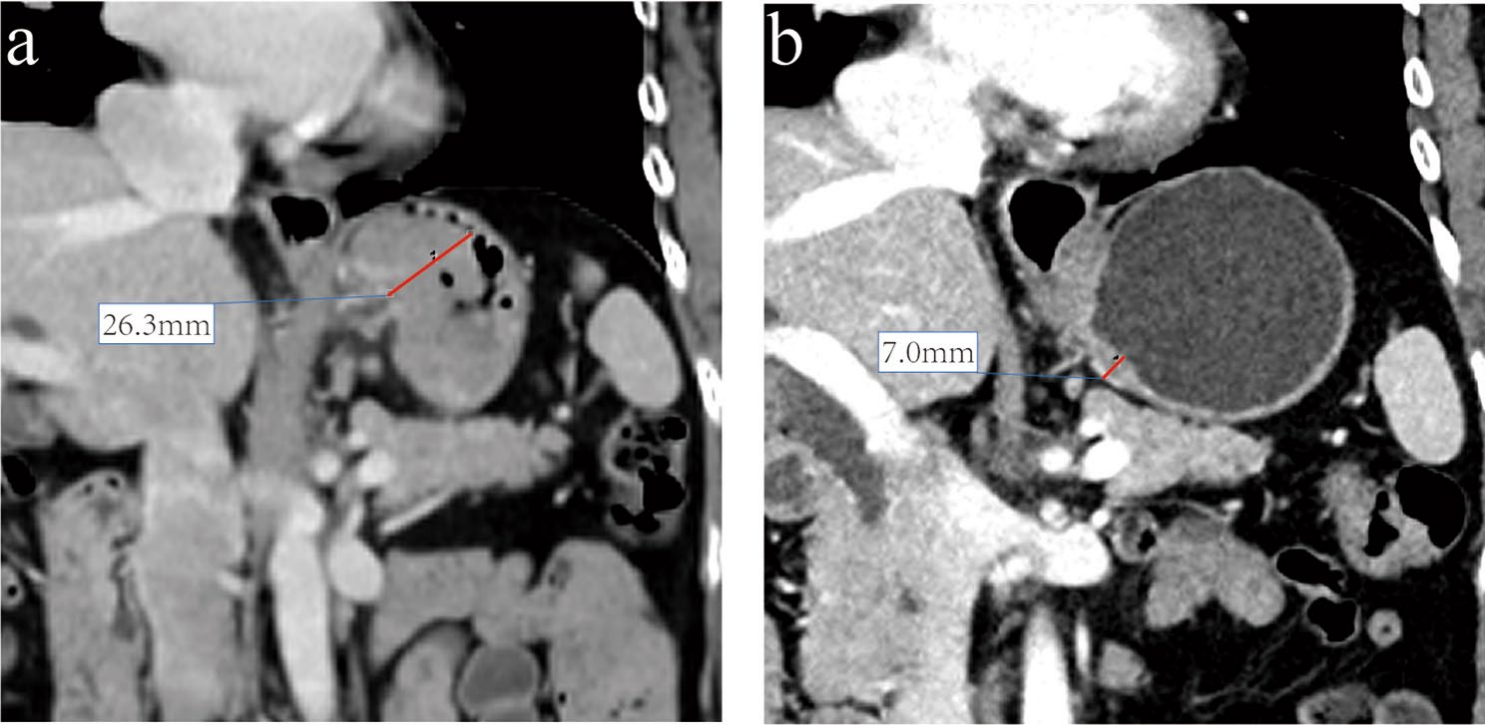
Female, 63 years, gastroscopy revealed a mass at the gastroesophageal junction. The biopsy pathology showed moderately differentiated adenocarcinoma with intestinal type of Lauren classification. The clinical stage was diagnosed as cT4aN1 by the preoperative CT examination. This patient received 3 cycles of XELOX chemotherapy and total gastrectomy. Postoperative pathology showed that the residual carcinoma in the original tumor bed was less than fibrous tissue hyperplasia. No metastases were observed in lymph nodes dissected in surgery (0/51). **A**: the baseline coronal CT image showed the gastric wall was significantly thickened. **B**: the coronal CT image after chemotherapy showed the primary tumor thickness shrunk

The CT features observed and measured are as follows: (a) the thickness of the primary lesion was measured after the layer with the largest tumor thickness was found in the cross-sectional, coronal, and sagittal planes, and the maximum value of the three was considered the tumor thickness (Fig. 1); (b) CT attenuation values of the primary lesions in the arterial and venous phase were measured by delineating the region-of-interest (ROI) at the level with the largest tumor thickness selected in (a) to increase reproducibility, and the areas of liquefaction and necrosis caused by chemotherapy on an arterial phase image were avoided as much as possible; and (c) the largest regional lymph nodes were selected to measure their long diameters, short diameters, and CT attenuation values. All measurements after NAC were kept at the same position. The ratios of reduction were calculated using the following formula: Rate value = (value before – value after)/value before × 100%.

### Statistical analyses

Quantitative and categorical data on the clinicopathological characteristics of the two groups were depicted as mean ± standard deviation and frequency (%), respectively. Independent-sample t-test was performed to compare quantitative variables. Chi-square test was used to determine differences in categorical variables. Univariate logistic regression analysis was performed on 16 CT features, from which the statistically significant features tested were included on multivariate logistic regression model to construct the regression equations. Receiver operating characteristic (ROC) curve analysis was used to evaluate the predictive accuracy of the CT features in differentiating responders from non-responders. Utility was evaluated using a decision curve analysis (DCA). We used SPSS 26.0 software (IBM Corp, NY, USA) and R version 4.1.2 software (The R Foundation for Statistical Computing, Vienna, Austria) for statistical analyses. A two-sided P < 0.05 was considered statistically significant.

## Results

### Clinicopathological characteristics of the patients

In this study, we included 97 patients (69 [71.1%] men; median [range] age, 60 [26–75] years) with locally advanced GC. The NAC regimens included XELOX (89 cases, oxaliplatin plus capecitabine) and SOX (8 cases, S-1 plus oxaliplatin). The mean number of treatment cycles was 3 (range, 2–4). In view of the obvious CT manifestations of locally advanced GC, all primary lesions found on gastroscope were successfully detected on CT.

On response evaluation following NAC, we identified 66 (68.0%) responders and 31 (32.0%) non-responders. The association between the patients’ characteristics and the treatment response to NAC is summarized in Table 1. The analyses showed that no characteristic was significantly associated with the treatment response, including age, sex, tumor location, differentiation grade, Lauren classification, and Borrmann type. At the same time, we found that the pathological T and N stage after NAC significantly differed between the two groups.

**Table 1:** Association of clinicopathological characteristics with treatment response

Characteristic	Responders (n = 66)	Non-responders (n = 31)	*P* value
Age	57.56 ± 10.75	57.03 ± 12.46	0.831
Sex			0.980
Male	47 (71.2%)	22 (71.0%)	
Female	19 (28.8%)	9 (29.0%)	
Tumour location			0.084
Antrum of the stomach	25 (37.9%)	5 (16.1%)	
Body of the stomach	6 (9.1%)	6 (19.4%)	
Cardia of the stomach	32 (48.5%)	17 (54.8%)	
The whole stomach	3 (4.5%)	3 (9.7%)	
Differentiation grade			0.256
G2	15 (22.7%)	4 (12.9%)	
G3	51 (77.3%)	27 (87.1%)	
Lauren classification			0.315
Intestinal	25 (37.9%)	8 (25.8%)	
Diffuse	28 (42.4%)	13 (41.9%)	
Mixed	13 (19.7%)	10 (32.3%)	
Borrmann type			0.199
II	11 (16.7%)	2 (6.5%)	
III	48 (72.7%)	28 (90.3%)	
IV	7 (10.6%)	1 (3.2%)	
Pathological T stage after NAC			0.002*
ypT0	8 (12.1%)	0 (0.0%)	
ypT1-2	17 (25.8%)	1 (3.2%)	
ypT3-4	41 (62.1%)	30 (96.8%)	
Pathological N stage after NAC			< 0.001*
ypN0	31 (47.0%)	2 (6.5%)	
ypN+	35 (53.0%)	29 (93.5%)	

Age and Sex are shown as median (range); other data are shown as number (percentage). *P* value is derived from the t-test (for quantitative variables) and Chi-square test (for categorical variables) analyses between clinical characteristics and treatment response

*NAC* neoadjuvant chemotherapy

*P value < 0.05

### Univariate logistic regression analysis

We analyzed 16 predictive features on univariate logistic regression. From these features, 11 were closely related to the occurrence of response after NAC and were screened out. Among the 11 features, post-NAC primary tumor thickness, post-NAC short-diameter of the largest lymph node, and post-NAC ratio of short-diameter to long-diameter of the largest lymph node were risk factors, while the rest were protective factors. The data are listed in Table 2.

**Table 2:** Univariate logistic regression analysis of CT features to predict response to NAC

CT features	OR	95% CI	*P* value
Pre-NAC primary tumor thickness (mm)	1.018	0.951–1.090	0.611
Post-NAC primary tumor thickness (mm)	1.191	1.084–1.308	< 0.001*
Pre-NAC short-diameter of largest lymph node (mm)	0.979	0.910–1.052	0.562
Post-NAC short-diameter of largest lymph node (mm)	1.107	1.000–1.224	0.049*
Pre-NAC long-diameter of largest lymph node (mm)	0.982	0.935–1.032	0.477
Post-NAC long-diameter of largest lymph node (mm)	1.042	0.970–1.118	0.260
Pre-NAC ratio of short-diameter to long-diameter of largest lymph node	0.449	0.013–15.828	0.660
Post-NAC ratio of short-diameter to long-diameter of largest lymph node	29.109	1.071–791.091	0.045*
Reduction in ratio of short-diameter to long-diameter of largest lymph node	0.018	0.001–0.490	0.017*
Ratio of primary tumor thickness reduction	0.000	0.000–0.001	< 0.001*
Ratio of reduction in short-diameter of largest lymph node	0.020	0.002–0.192	0.001*
Ratio of reduction in long-diameter of largest lymph node	0.058	0.007–0.510	0.010*
Ratio of reduction of primary tumor attenuation in arterial phase	0.013	0.002–0.092	< 0.001*
Ratio of reduction of largest lymph node attenuation in arterial phase	0.036	0.007–0.179	< 0.001*
Ratio of reduction of primary tumor attenuation in venous phase	0.028	0.005–0.168	< 0.001*
Ratio of reduction of largest lymph node attenuation in venous phase	0.064	0.014–0.288	< 0.001*

*CT* computed tomography, *NAC* neoadjuvant chemotherapy

*P value < 0.05

### Multivariate logistic regression analysis

**Fig. 2:**
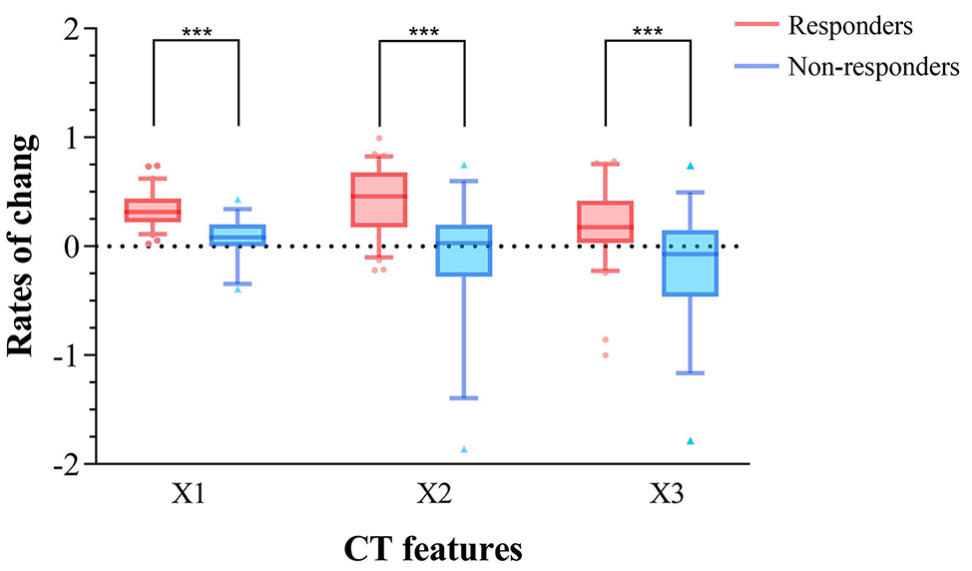
The distribution of computed tomography (CT) parameters in responders and non-responders. ***p < 0.001. X1: ratio of primary tumor thickness reduction; X2: ratio of reduction of primary tumor attenuation in arterial phase; X3, ratio of reduction of largest lymph node attenuation in venous phase

The 11 features that were statistically significant on univariate analyses were analyzed on multivariate logistic regression analysis. As a result, 3 independent factors were included in the logistic regression equation, the maximum contribution was ratio of primary tumor thickness reduction (X1), followed by ratio of reduction of primary tumor attenuation in arterial phase (X2), and ratio of reduction of largest lymph node attenuation in venous phase (X3) (Table 3). The distribution of these 3 CT features in responders and non-responders is shown in Fig. 2.

**Table 3:** Multivariate logistic regression analysis of CT features to predict response to NAC (Forward, LR, a = 0.05)

CT features	Partial regression coefficient	Standard error	OR (95% CI)	*P* value
Ratio of primary tumor thickness reduction (X1)	-13.318	3.751	0.000 (0.000–0.003)	< 0.001*
Ratio of reduction of primary tumor attenuation in arterial phase (X2)	-5.306	1.621	0.005 (0.000–0.119)	0.001*
Ratio of reduction of largest lymph node attenuation in venous phase (X3)	-2.694	1.133	0.068 (0.007–0.623)	0.017*
Constant	2.957	0.947	19.244	0.002

*CT* computed tomography, *NAC* neoadjuvant chemotherapy

*P value < 0.05

**Table 4:** ROC curve analysis of CT features to predict response to NAC

CT features	AUC	95% CI	Cutoff value
Ratio of primary tumor thickness reduction (X1)	0.884	0.815–0.954	0.230
Ratio of reduction of primary tumor attenuation in arterial phase (X2)	0.837	0.753–0.922	0.389
Ratio of reduction of largest lymph node attenuation in venous phase (X3)	0.766	0.663–0.870	0.340
Multivariate logistic regression model (*p* = 1/(1 + e^(−2.957+13.318*X1+5.306*X2+2.694*X3)^))	0.955	0.911–0.998	0.369

*ROC* Receiver operating characteristic, *CT* computed tomography, *NAC* neoadjuvant chemotherapy, *AUC* area under the curve

**Fig. 3:**
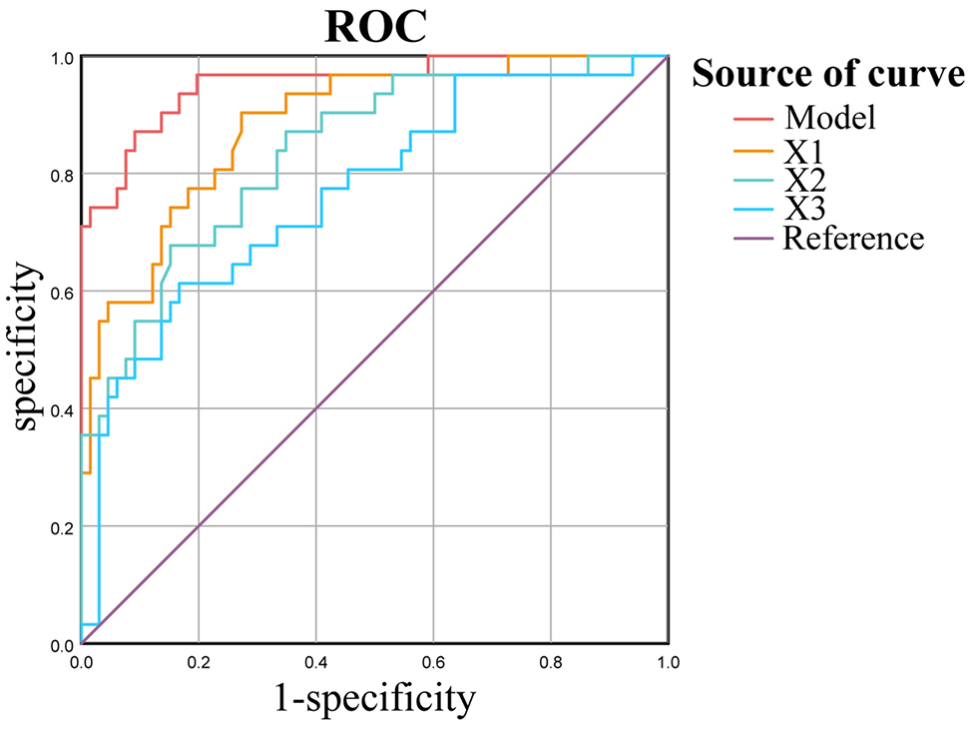
Receiver operating characteristic (ROC) curves of computed tomography (CT) features for predicting response to neoadjuvant chemotherapy (NAC). X1: ratio of primary tumor thickness reduction; X2: ratio of reduction of primary tumor attenuation in arterial phase; X3: ratio of reduction of largest lymph node attenuation in venous phase

**Fig. 4:**
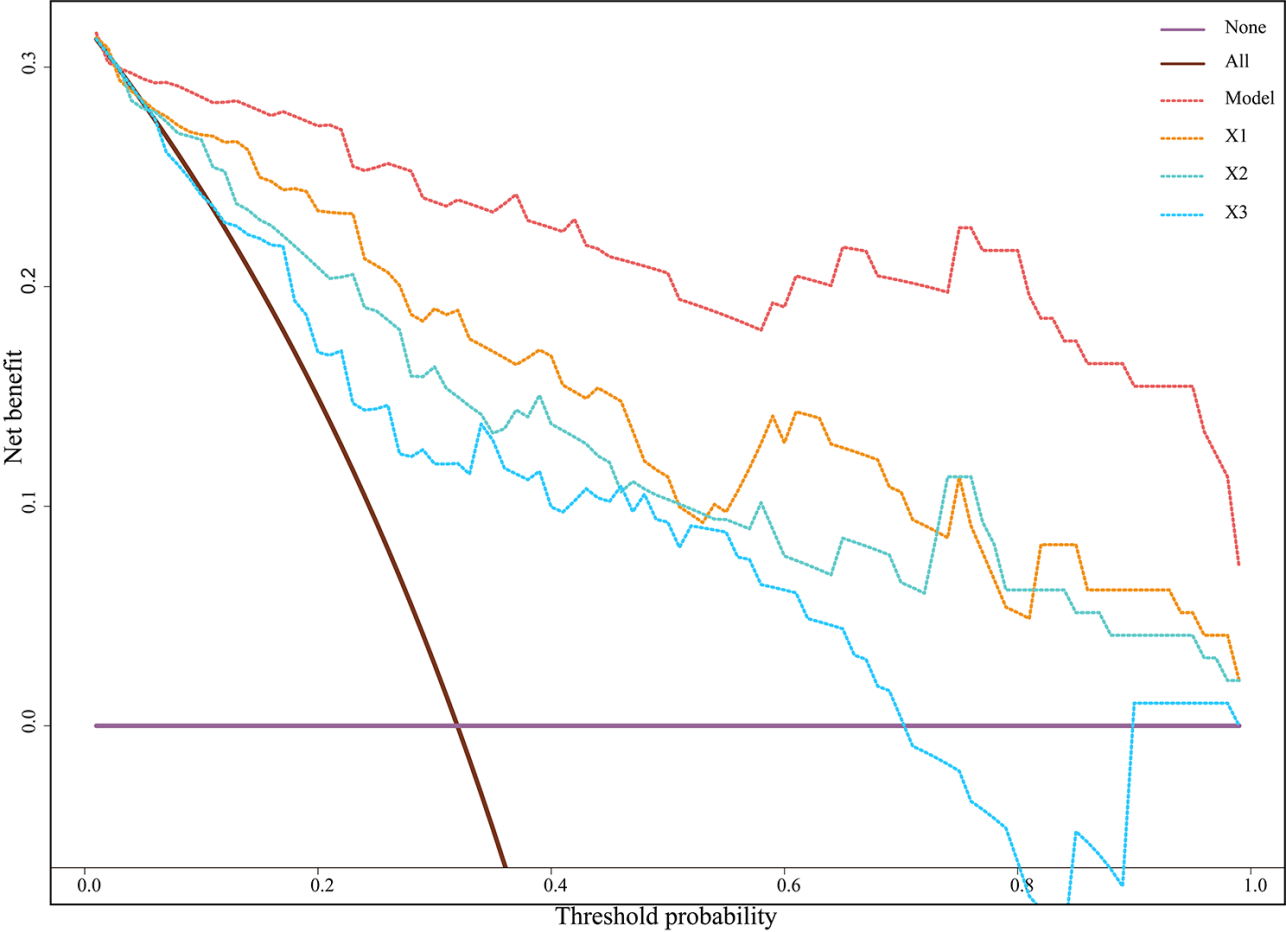
Decision curve analysis (DCA) of computed tomography (CT) features for predicting response to neoadjuvant chemotherapy (NAC). X1: ratio of primary tumor thickness reduction; X2: ratio of reduction of primary tumor attenuation in arterial phase; X3: ratio of reduction of largest lymph node attenuation in venous phase

Based on the logistic regression equation, a multivariate prediction model that calculated the risk of non-response after NAC was established, *p* = 1/(1 + e^(−2.957+ 13.318*X1+5.306*X2+2.694*X3)^). The closer the *p*-value was to zero, the higher the probability of response, and the closer it is to one, the higher the probability of non-response. With the prediction probability of 0.5 as the cutoff value, the correct rate, sensitivity and specificity were 88.7%, 77.4%, and 93.9%, respectively. The area under the ROC curve (AUC) of the multivariate logistic regression model for discriminating responders from non-responders was large, being 0.955 (95% CI, 0.911–0.998) (Table 4; Fig. 3). Moreover, the DCA in Fig. 4 showed that the net clinical benefits of the multivariate model significantly outperformed the three individual factors at all threshold ranges.

## Discussion

The present retrospective study confirmed that CT features were able to bridge imageology and histopathology by using postoperative pathology as a reference outcome to identify responders to NAC. The logistic regression equation generated based on the 3 selected CT features showed a good predictive performance. This equation is applicable to predict response to NAC and offer evidence-based guidance for timely and reasonable individualized multidisciplinary treatment decision-making in patients with locally advanced GC.

Since the stomach is a hollow organ, the stability of the long-diameter measurement of the primary lesion of GC is greatly affected by gastric filling and peristalsis. The RECIST 1.1 criteria [10, 11] does not recommend measuring the long-diameter of GC as a reference for therapeutic evaluation. Therefore, we did not include the long-diameter of primary lesions as a potential CT feature. However, the guidelines of the Chinese Society of Clinical Oncology (CSCO) [12] stated that the thickness of the primary lesions could be used as a reference. Although previous studies [13–16] have reported the use of CT features to predict pathological response, these studies had small sample sizes and did not focus on the role of GC thickness. On multivariate analysis of this study, the ratio of tumor thickness reduction was found to be a strong independent predictor of response, which provided theoretical basis for its practicability. It should be noted that the difficulty in clinical application of this parameter lies in the quality control of thickness measurement. Yang et al. [17] investigated whether the measurement of GC thickness was affected by the degree of gastric filling and found that lesion thickness was similar in newly diagnosed patients prior to and following gastric filling, but significantly differed in patients on reexamination. This suggests that maintaining a similar degree of gastric filling and the same measurement position at re-examination as at the first examination facilitates accurate therapeutic evaluation.

Chemotherapy-induced tumor ischemia, necrosis, and liquefaction lead to changes in tumor density [8, 18], and these intra-tumor changes may alter the morphological changes of the tumor. This suggests that paying attention to the density changes while monitoring the thickness reduction can provide more predictive information for therapeutic evaluation. For this reason, Choi criteria [18] were proposed and first applied in gastrointestinal stromal tumors. Liu et al. [8] defined adapted Choi criteria as a reduction of at least 10% in the sum of short-diameters or 15% in mean volumetric attenuation of target lymph nodes, and found that tumor attenuation can be used as a supplement to tumor size. This adapted Choi criteria could be helpful to predict the survival of patients with locally advanced GC. In this study, we found that the status of the primary tumor attenuation in arterial phase and the status of the largest lymph node attenuation in venous phase were effective predictors of responders. The attenuation in arterial phase can reflect the enhancement characteristics of the gastric wall and GC tissue [19], indicating the capillary density and blood supply of the primary tumor. However, the venous phase focuses on the balance and retention of contrast agent in the interstitial space, which is beneficial to the detection of lymph node metastasis [20]. We consider that the different observed phases of the primary tumor and lymph nodes may be due to their different enhancement characteristics. On the other hand, consistent with the basic principle of Choi criteria, our results showed that a higher reduction in attenuation of the primary tumor and the largest lymph node was associated to a better pathological response. In contrast to this, a recent study published by Cihan et al. [21] had an opposite conclusion. They found that a higher decrease in CT attenuation was associated to a poor pathological response to perioperative chemotherapy (FLOT regimen) in patients with GC and gastroesophageal junction adenocarcinoma. This difference may be related to their measurement method and the fact that patients with poor pathological responses in their study had higher attenuation values at diagnosis.

According to the RECIST 1.1 criteria, the target lesions of locally advanced GC are malignant lymph nodes with a short-diameter ≥ 15 mm only. That is, not all patients receiving NAC are considered to have evaluable target lesions. Although this greatly improves the specificity of screening for metastatic lymph nodes, it also ignores normal-sized metastatic lymph nodes and limits the application of RECIST in GC. Among the 97 patients in our study, 23 had a short-diameter of the largest lymph node ≥ 15 mm prior to treatment. However, more than 23 patients had lymph node metastases. Some studies suggest that using smaller lymph nodes as predictive criteria appears to be a viable way to screen for more positive lymph nodes. Dai et al. [22] used a long-diameter ≥ 8 mm as criterion to predict lymph node metastasis in GC and obtained a sensitivity and specificity of 79.6% and 78.8%, respectively. Saito et al. [23] used an optimal cutoff value for the short-diameter of 6 to 9 mm based on different histological types and different regions of lymph node location, with an accuracy of 71.1% and 76.6%, respectively to diagnose lymph node metastasis. Positive nodes were also present in lymph nodes ≤ 5 mm in size. Noda et al. [24] indicated that if all lymph nodes ≤ 5 mm were ignored, 37.8% of all metastatic lymph nodes would be missed. In order to improve the sensitivity, we selected the largest lymph node of all patients, namely the most representative lymph node, as target organs. In 96 of 97 patients, the short-diameter of the largest lymph node prior to treatment was ≥ 5 mm, and only 1 patient had the short-diameter of largest lymph node equal to 4 mm. On univariate logistic analysis, the rate of short-diameter reduction, long-diameter reduction, and reduction of the largest lymph nodes attenuation significantly differed between responders and non-responders. On final multivariate analysis, the ratio of reduction of largest lymph node attenuation in venous phase was an independent predictor of response.

At present, radiological evaluation of response to NAC is rapidly developing. The team of our center has recently proposed and externally validated a radiomics signature to identify responders to NAC using pretreatment enhanced CT images [25]. Nevertheless, the advantage of the prediction model in the present study over radiomics is the possibility to quickly, conveniently, and easily calculate the probability of pathological response using this regression equations without the need for complex algorithms and equipment. This makes this method possible to be used widely in the clinic.

This study has limitations. First, this was a single-center study, and validation in a larger prospective cohort is required to further confirm the clinical significance of these CT features given the heterogeneity of GC among different populations. Second, we did not analyze multi-dimensional indicators such as lesion area and lesion volume. To a certain extent, this may affect the predictive ability.

## Conclusion

We developed a logistic regression equation based on three CT features: the ratio of tumor thickness reduction, ratio of reduction of primary tumor attenuation in arterial phase, and ratio of reduction of largest lymph node attenuation in venous phase to differentiate between responders and non-responders to NAC. This prediction model could be used as a reference for oncologists to make effective personalized treatment decisions in a timely manner.

## Data Availability

The datasets analysed during the current study are available from the corresponding author upon reasonable request.
